# Malaria prevalence and risk factors among patients visiting Mizan Tepi University Teaching Hospital, Southwest Ethiopia

**DOI:** 10.1371/journal.pone.0271771

**Published:** 2022-07-28

**Authors:** Tadesse Duguma, Eyob Tekalign, Dassalegn Muleta, Asnake Simieneh

**Affiliations:** Department of Medical Laboratory Science, College of Health Science and Medicine, Mizan-Tepi University, Mizan-Aman, Ethiopia; Menzies School of Health Research, AUSTRALIA

## Abstract

**Background:**

Ethiopia is among sub-Saharan African countries with a high number of malaria cases each year, with most of the landmass favoring the breeding of the vectors. There have been extensive efforts to control and prevent the transmission of malaria, which is part of the country’s prevention-based health policy.

**Objective:**

This study aimed to determine malaria prevalence and associated risk factors among patients visiting Mizan-Tepi University Teaching Hospital (MTUTH).

**Materials and methods:**

A cross-sectional study was conducted from September to December 2021 among patients visiting MTUTH, Southwest Ethiopia. A pretested structured questionnaire was used to collect sociodemographic data, and a capillary blood sample was collected after obtaining written informed consent from the study participants. The data were entered into Epi-data manager (v4.0.2.101) and analyzed with SPSS version 25.0, with a P-value of < 0.05 set as a significance.

**Results:**

A total of 439 patients participated, of which 20.7% (91) were positive for malaria parasites, with a higher prevalence observed among the age group interval of 25–34 years (5.5%). Inadequate access to insecticide-treated bed net (ITN) 23.9% (105) and a low level of ITN usage 20.5% (90) were recorded. Patients living in areas of stagnant water were more likely to get infected with the malaria parasite (AOR = 16.191, 95% CI: 9.137, 28.692) compared to those who live away from stagnant water, and individuals living in houses not sprayed with insecticides were more susceptible to malaria infection (AOR = 0.215, 95% CI: 0.128, 0.360).

**Conclusion:**

The overall malaria prevalence in this study was 20.7% (91), which proves that malaria remains a major threat to the communities in the study area, with *Plasmodium falciparum* contributing to most of the cases. Improving the habits of ITN usage and indoor residual spray through health education may help to reduce the impact of malaria in the study area.

## Introduction

Malaria is a major public health problem that still results in illness and death. Globally, malaria cases increased from 227 million cases in 2019 to 241 million in 2020, reported 85 malaria-endemic countries [1]. In the last two years, global malaria case numbers have risen by 14 million cases and deaths by 47,000 due to disruptions during the coronavirus pandemic [2]. Malaria is one of the major diseases affecting people of low socioeconomic status in developing countries. The majority of the global malaria burden is in sub-Saharan Africa, with the highest global cases and deaths that can be observed from these regions, which accounted for the majority (95%) of these cases. Nigeria (27%), the Democratic Republic of the Congo (12%), Uganda (5%), Mozambique (4%), and Niger (3%) accounted for about 51% of all cases globally [3]. It hurts people’s health as well as the economic development of many developing countries, particularly in sub-Saharan Africa [1,4,5].

The most common causes of human malaria are four species, namely, *Plasmodium vivax*, *Plasmodium falciparum*, *Plasmodium malariae*, *Plasmodium ovale*, and sometimes a fifth species, *Plasmodium knowlesi*, which is zoonotic [6]. The most prevalent and pathogenic malaria parasite, most commonly associated with severe illness and death, accounting for 99.7% of malaria cases in the WHO African region, 70.9% from Central America, 20.8% from South America, and 4.8% from Asia, is *P*. *falciparum* [7]. *Plasmodium falciparum* is responsible for most human infections, but *Plasmodium vivax* has the largest geographic reach [8]. Ethiopia, as one of those countries, suffers from this disease, which is posing a problem to the health and economy of the country. The distribution of malaria in Ethiopia varies depending on climate, rainfall patterns, and altitude [9]. A 2021 systematic review showed that the highest malaria prevalence in the Southern Nations, Nationalities, and Peoples’ Region (SNNPR) (16.17%), followed by Oromia Regional State (13.11%), and Amhara Regional State (12.41%) [10].

Ethiopia is among the few African countries where both *Plasmodium falciparum* and *Plasmodium vivax* species are co-endemic in substantial proportions [11].

The *Anopheles* mosquito serves as the primary vector for malaria transmission in Ethiopia. *Anopheles arabiensis* is the main vector, followed by *Anopheles phronesis*, *Anopheles funestus*, and *Anopheles nili*, which play a role as secondary vectors [12]. The risk of infection is determined by the number and species of mosquitoes present in a given area and the climate and geography [13].

Ethiopia has three climatic zones, namely "Kola", "Woina Dega", and "Dega", the first two having favorable characteristics (i.e., warmer and humid climate conditions) for malaria endemicity [14]. The ‘Kola,’ or hot zone with an altitude below 1500 meters, has seasonal malaria transmission with moderate to high endemicity (46% of the territory), whereas the "Woina Dega," or temperate zone, has unstable malaria transmission characterized by sporadic outbreaks caused by sudden climatic changes such as heavy rain or clouds. The "Dega," or cold zone (8% of the territory), is a climatic area 2500 above sea level where malaria transmission is not common [14]. Nearly 75% of the country’s landmass is conducive to malaria transmission, putting 68% of the population at risk of contracting the disease [15,16].

In Ethiopia, malaria transmission varies from one season to another due to variation in the altitude of areas with a relatively longer duration of transmission in lowland areas, river basins, and valleys [17]. There are two malaria transmission seasons during which the vectors are abundant: from September to December and the early rainy season of April to May, which overlaps with the major harvesting seasons [17]. Malaria outbreaks are common in some of Ethiopia’s highland or highland fringe areas, mainly 1,000 to 2,000 meters above sea level [18]. Climate changes contributing to malaria transmission dynamics include alternations in one or more climate variables, including temperature, precipitation, wind, and sunshine [16,19]. Estimates from 2016 indicate there were 2.9 million cases and 4,782 related mortalities, and have been reported per year, and morbidity and mortality rates dramatically increase during epidemics [15]. Despite key achievements and progress in reducing the burden of the disease, malaria remains a major health problem for the public. It is among the 10 top leading causes of illness and death in large segments of the population, including children under the age of five and adults [20]. Ethiopia is still one of the countries with a high malaria burden, and this was evidenced by the deaths of people in the study area, including children and pregnant women, which resulted in reduced working capacity and other day-to-day activities of the community. This study quantifies the malaria burden and therefore could serve as baseline data for the concerned bodies to boost preventative and control methods to reduce and, in the long run, eliminate the disease’s health impact.

## Materials and methods

### Ethical statement

Ethical approval was obtained from the research committee of the college of health sciences and medicine of Mizan-Tepi University before the commencement of the study. Written informed consent was obtained from all participants except for infants, children, and minors, whose consent was obtained through their parents/guardians after explaining the study’s purpose, possible risks, and benefits. Moreover, participants were assured that participation is entirely voluntary and can be withdrawn at any time during the data collection process.

### Study area and period

The study was conducted between September and December 2021 at Mizan-Tepi University Teaching Hospital in Mizan-Aman town, Benchi-Sheko zone, SNNPR region, Southwest Ethiopia, which is located 565 kilometers from Addis Ababa, the capital of the country. The research site is located in a region of the country known for its warm and humid climate, with coordinates of 7°0′N 35°35′E and an elevation of 1451 meters. Based on the 2012 census conducted by the central statistical agency, the total population of Mizan-Aman town was 63,193, of whom 32,596 were male and 30,596 were female. Mizan-Aman town is the administrative town of the Benchi-Sheko zone, one of 13 zones of SNNPR, and has 12 woredas (districts).

### Study design and population

An institutional-based cross-sectional study was employed.

All patients who visited MTUTH during the study period were the source population, while all malaria-suspected patients who were sent to the laboratory of the hospital for blood film examination were taken as a study population.

Those willing to participate and provide blood samples were recruited as the study subjects. Anyone who took antimalarial drugs and antibiotics in the last month was not included in the study.

### Sample size and sampling technique

The sample size was calculated using a single population proportion formula, with a 95% confidence interval and an estimated malaria prevalence rate of 11% (based on a study from Jimma zone, Southwest Ethiopia) [14]. Based on these considerations, the sample size was calculated using the following formula:

$$\text{n}=\frac{{\left(Z\alpha /2\right)}^{2}p\left(1-P\right)}{d^{2}}$$

= (1.96)2 0.11(1−0.11)/0.032 = 417.880≈418

After adding a 10% buffer for possible non-response,

the final sample size became 418 + 418(0.1) = 459.8≈460

Where n = minimum sample size

P = estimated malaria prevalence rate of 11% (study from the Jimma Zone, Southwest Ethiopia)

d = error margin (3%)

(Zα/2)) 2 = the standard normal variable.

The study participants were recruited by a systematic random sampling technique, considering the case flow in the hospital from September to December of the previous year.

### Data collection instruments and procedures

Structured questionnaires adapted from related literature [21] that contain both socio-demographic and risk factor variables were used to collect the data. Before blood sample collection, the finger was cleaned with 70% alcohol-moistened cotton. A drop of blood, approximately 50 μL (capillary blood from fingertip) by finger prick, was collected from each study subject, and both thick and thin films were made according to the standard operating procedure.

### Data collection process and management

Before the start of the data collection process, two-day training on the objective of the study was given to the data collectors (interview and blood sample). Written informed consent was obtained from the study participants (adults); and for infants, children, and minors, consent was obtained by communicating with their guardian/caregiver, or parents. Three medical laboratory technicians were recruited for data collection, along with two supervisors to facilitate the data collection processes. The blood films (thick and thin) were stained using a 10% Giemsa working solution and examined microscopically using a 100X oil immersion objective to detect malaria parasites.

### Data quality assurance

The data collection procedures, tools used, and how to handle ethical issues were discussed with the data collectors. A pretest was conducted on 5% of the sample size before the commencement of the main study in Tepi general hospital, which is located 50 kilometers away from the study area. The questionnaire was translated into the respondent’s language during data collection. Regular supervision by the supervisors and the principal investigators was conducted to ensure that all the necessary data was properly collected.

### Statistical analysis

Epi-data manager (v4.0.2.101) was used to enter data, and SPSS version 25.0 was used for analysis. Descriptive statistics and both bivariate and multivariable logistic regression were performed to assess the existence of an association between the outcome variable and the risk factors.

## Results

### Socio-demographic characteristics of the study subjects

Of the total 439 patients who participated in this study, a significant number of participants (91) were infected with malaria parasites. This study included 208 males and 231 females. Malaria was reported in all age groups [Table 1] but the infection rate was highest in the 25–34 age group (5.5%). Almost half of the study participants were illiterate. Of the different occupations included in the study, the most cases were among housewives (35) and students (22) [Table 1].

**Table 1:** Malaria prevalence by sex, age, educational level, and occupation among patients undergoing blood film examination at MTUTH in 2021 (n = 439).

Socio-demographic variables		Malaria		
		Positive N (%)	Negative N (%)	Total N (%)
Sex	Male	43(9.8)	165(37.6)	208(47.4)
	Female	48(10.9)	183(41.7)	231(52.6)
Age in year/s	0–4	6(1.4)	49(11.2)	55(12.5)
	5–14	11(2.5)	63(14.4)	74(16.9)
	15–24	15(3.4)	76(17.3)	91(20.7)
	25–34	24(5.5)	86(19.6)	110(25.1)
	35–44	20(4.6)	49(11.2)	69(15.7)
	45–55	13(3)	23(5.2)	36(8.2)
	>55	2(0.5)	2(0.5)	4(0.9)
Educational status	Illiterate	53(12.1)	166(37.8)	219(49.9)
	Literate	38(8.6)	182(41.5)	220(50.1)
Occupational status	Farmer	10(2.3)	41(9.3)	51(11.6)
	Merchant	0(0)	15(3.4)	15(3.4)
	Government Employee	7(1.6)	29(6.6)	36(8.2)
	Student	22(5)	113(25.7)	135(30.8)
	Housewife	35(8)	89(20.3)	124(28.2)
	Daily worker	15(3.4)	45(10.3)	60(13.7)
	Other	2(0.5)	16(3.6)	18(4.1)
**Total**		91(20.7)	348(79.3)	439(100)

### Malaria prevalence

**Fig 1:**
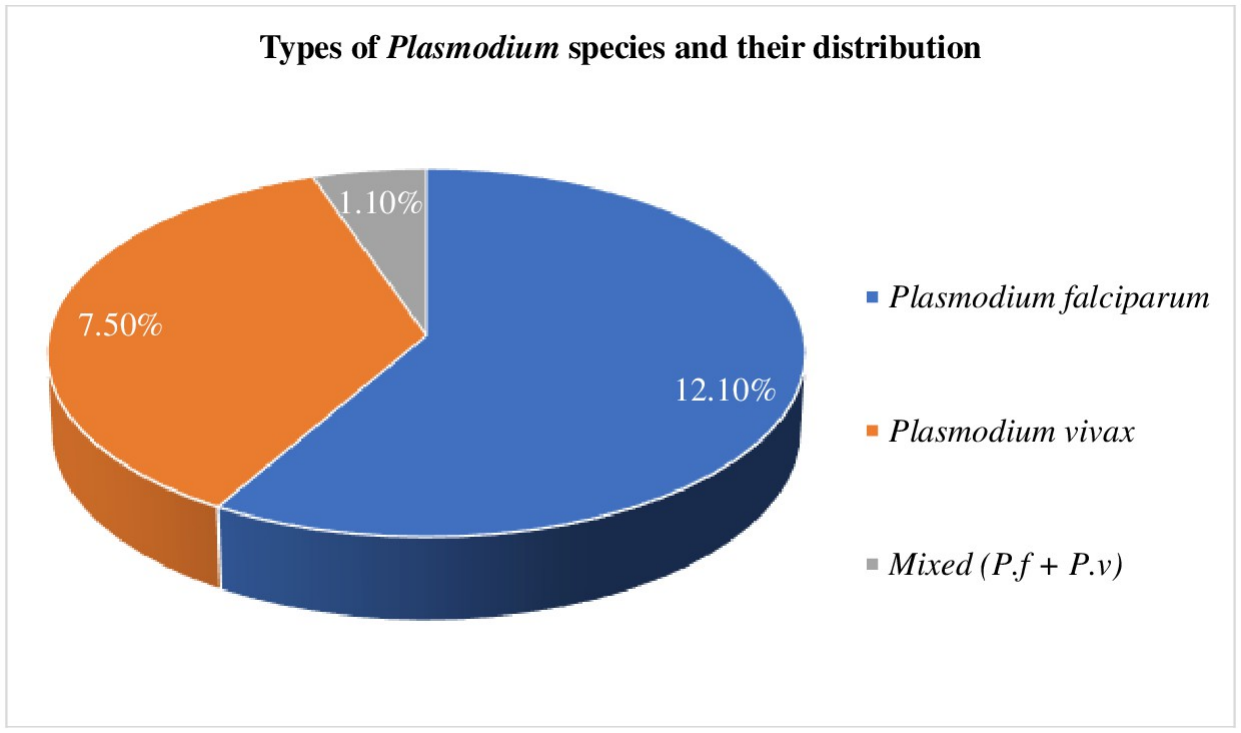
*Plasmodium* species and their distribution.

A total of 460 study participants were recruited for this study, of which only 439 participated in the interview and provided blood samples (a non-response rate of 4.6%). Of those tested 20.7% (91) were found to be infected with malaria parasites, of which 12.1% (53), 7.5% (33), and 1.1% (5) were *Plasmodium falciparum*, *Plasmodium vivax*, and mixed infection, respectively [Fig 1].

### Malaria prevalence concerning climate conditions

Of the total study participants, 349 (79.5%), 68 (15.5%), and 22 (5.0%) were from warmer "Kolla" conditions, while medium "Woina Dega" and colder "Dega" conditions had a respective malaria prevalence of 17.8%, 2.3%, and 0.7%, respectively.

### Possible risk factors for malaria infection

Data was collected from 439 study participants to investigate the contributions of possible risk factors to the prevalence of malaria. The presence of stagnant water near the residence, a house sprayed with insecticide, a history of anti-malarial treatment, residence type, and last night’s ITN usage were among the possible risk factors assessed. Accordingly, more than three-fourths of respondents did not have access to it, and more than two-thirds of the participants reported that their houses did not undergo IRS. This finding indicates lack of access to ITNs and inadequate insecticide spray contributed to the malaria prevalence observed in this study. This study showed not only inadequate access to ITNs 23.9% (105) but also a low level of ITN usage at 20.5% (90). Some risk factors were associated with malaria infection, presence of stagnant water near residential (AOR = 16.191, 95% CI: 9.137, 28.692), houses sprayed with insecticide (AOR = 1.799, 95% CI: 1.054, 3.073), history of anti-malarial treatment (AOR = 0.215, 95% CI: 0.128, 0.360), and residence respectively (AOR = 1.971, 95% CI: 1.034, 3.756), , , and [Table 2].

**Table 2:** Bivariate and multivariable analysis of factors associated with malaria infection concerning ITN availability, ITN usage, presence of stagnant water, indoor residual spray, history of malaria treatment, and residence at MTUTH, 2021 (n = 439).

Variables		Malaria			OR (95% CI)	
		Positive N (%)	Negative N (%)	Total N (%)		
					COR	AOR
ITN Availability	Yes	15(3.4)	90(20.5)	105(23.9)	1.00	1.00
	No	76(17.3)	258(58.8)	334(76.1)	0.566(0.309,1.035)	1.949(0.972,3.907)
Last night’s ITN usage	Yes	17(3.8)	73(16.6)	90(20.5)	1.00	1.00
	No	74(16.9)	275(62.7)	349(79.5)	0.865(0.481,1.556)	0.821(0.415,1.622)
Presence of Stagnant water	Yes	72(16.4)	66(15)	138(31.4)	0.062(0.035,0.109) *	16.191(9.13,28.692) *
	No	19(4.3)	282(64.2)	301(68.6)	1.00	1.00
House sprayed with insecticide	Yes	21(4.8)	122(27.8)	143(32.6)	1.00	1.00
	No	70(15.9)	226(51.5)	296(67.4)	0.556(0.325,0.949) *	1.799(1.054,3.073) *
History of anti-malarial treatment	Yes	43(9.8)	55(12.5)	98(22.3)	1.00	1.00
	No	48(10.9)	293(66.7)	341(77.7)	4.772(2.888,7.887) *	0.215(0.128,0.360) *
Residence	Urban	30(6.8)	193(44.0)	223(50.8)	1.00	1.00
	Rural	61(13.9)	155(35.3)	216(49.2)	0.395(0.243, 0.642) *	1.971(1.034,3.756) *
Total		91(20.7)	348(79.3)	439(100)		

**Abbreviations**: AOR, adjusted odds ratio; COR, crude odds ratio; OR, odds ratio,

(*) indicates significance at p<0.05,

"1.00 represents" reference category during analysis.

## Discussion

This study included 208 males and 231 female participants with a mean age of 24.16 (SD ± 14.931) and a median age of 24.00 years. Malaria parasite infection was recorded in 20.7% (91) of them, with the highest prevalence due to *Plasmodium falciparum* 12.1%, followed by *Plasmodium vivax* 7.5%. A study from Butajira, south-central Ethiopia, found a similar prevalence of *Plasmodium falciparum* (12.4%) [22]. The findings from this study revealed that the prevalence of malaria was higher among females (10.9%) than males (9.8%), which is in line with a previous study conducted in Ghana [23]. In this study, the prevalence of malaria was found to be higher in the 25–34 age group (5.5%) compared to other age groups, which is in line with a previous study conducted in Ghana that reported the prevalence of malaria to be higher in similar age groups [23]. The prevalence of malaria among illiterate was higher at 12.1% compared to literate (8.6%). The reason for these variations may be due to differences in the level of understanding of the preventive and control methods among the study participants. Patients with different occupations participated in the study, the highest prevalence of malaria was found among (8%) housewives and students (5%). Out of the positive cases, 226 (51.5%) study participants responded that their house was not sprayed with insecticide/chemical compared to those whose house was sprayed (4.8%), which may explain the relatively high malaria prevalence (15.9%) in this study. Malaria prevalence was found to be higher in rural residents (13.9%) as compared to urban residents (6.8%). This could be due to low exposure to health education and accessibility of the media for communication in rural communities. Although the study participants agreed that "the usage of it is a powerful vector control tool for the prevention of malaria transmission and hence reduces the prevalence of the disease elsewhere in the country where malaria is endemic," only 23.9% of them had ITNs in their houses, and ITN ownership by itself is not a guarantee of its usage. This is demonstrated by three-fourths (79.5%) of the study participants not using their bed nets [Table 2]. ITN use in this study was similar to what was found in a study in Kenya: approximately 92.11% of mosquito bed net usage, and malaria prevalence was observed to be lower among households that used ITNs (8.05%) compared to those that did not use them (23.11%) [24]. In our study, the use of ITNs appears to be one of the most effective malaria prevention methods. The prevalence of malaria among those who were not using ITNs 16.9% (74) was higher as compared to those who used them 3.8%(17). More than half of the study participants responded that they had no ITN in their houses, which is evidenced by the high malaria infection observed among those ITN non-users. Seventy-two (16.4%) patients whose blood film examination revealed the presence of malaria parasites responded that there was stagnant water near their homes. The malaria prevalence found in this study 20.7% (91/439) was lower compared to studies conducted in different parts of the world, including various areas of Nigeria (35.7%, 419/1173) [25], India (36.6%) [26], Malaysia (33.6%, 410/1222) [27], and Kenya (28%, 325/1158) [28]. It is also comparable to findings from Rwanda (22.8%, 175/769) [29], East Shewa Ethiopia (20.5%, 170/830) [30], Arba Minch South Ethiopia (22.1%, 60/271) [31], and much higher compared to studies from North-West Ethiopia (7.3%, 296/4077), (3.5%, 26/735) [32,33]. These variations may be due to the differences in the geographical location and climate conditions of the study areas. The result of this study showed that malaria is still a serious public health concern in different locations of the country, so the information obtained from this particular study can be used to devise means (control and prevention strategies) to prevent further suffering of people from this disease.

## Limitations

The findings of this study could have been better if testing had been done using advanced molecular techniques like polymerase chain reaction (PCR) and loop-mediated isothermal amplification (LAMP), which have higher detection abilities compared to light microscopy.

## Conclusions

The overall malaria prevalence in the study was 20.7% (91), which proves that malaria remains a major health problem in the area with *Plasmodium falciparum* as the predominant species in the study area. Improving the habits of ITN usage in the community through health information dissemination may help to prevent transmission.

## Supporting information

S1 Data set (SAV)

S1 File (DOCX)

## Abbreviations

AOR: adjusted odds ratio
COR: crude odds ratio
ITNs: insecticide-treated nets
LAMP: loop-mediated isothermal amplification
OR: Odds ratio
PCR: Polymerase chain reaction
SPSS: Statistical packages for social sciences
WHO: World health organization

## References

[1] World Health Organization. "World Malaria Report 2020". 20 years of global progress and challenges, 2020. https://www.who.int/publications-detail-redirect/97892400157919789240015791-eng.pdf.

[2] World Health Organization, "World Malaria Report 2021". Available online at ISBN 978-92-4-004049-6 (electronic version).

[3] Badmos AO, Alaran AJ, Adebisi YA, Bouaddi O, Onibon Z, Dada A. Others sub-Saharan African countries can learn from malaria elimination in China. Tropical Medicine and Health. 2021;49(1):1–6. 34689839 10.1186/s41182-021-00379-z PMC8542407

[4] Barofsky J, Claire C, Tobenna A, and Farshad F. The economic effects of malaria eradication: Evidence from intervention in Uganda: Program on the Global Demography of Aging Working Paper. 2011; (70). http://www.hsph.harvard.edu/pgda/working.htm.

[5] Castillo R, McIntyre M, and Barnes KD. The household burden of malaria in South Africa and Mozambique: is there a catastrophic impact? Tropical medicine & international health. 2008; 13(1): 108–122. Available online at doi: 10.1111/j.1365-3156.2007.01979.x 18291009

[6] Krief S, Pacheco AE, Mugisha M A. et al. On the diversity of malaria parasites in African apes and the origin of *Plasmodium falciparum* from Bonobos. PLoS pathogens. 2010; 6(2). Available online at doi: 10.1371/journal.ppat.1000765 20169187 PMC2820532

[7] Mace KE, Arguin PM, Tan KR. Malaria surveillance United States, 2015. MMWR Surveillance Summaries. 2018;67(7):1. Available online at doi: 10.15585/mmwr.ss6707a1 29723168 PMC5933858

[8] World Health Organization. World malaria report: Geneva. 2018. Available online at (Google Scholar).

[9] Legesse Y, Ayalew T, Tefera B, and Kora T. Knowledge, attitude, and practice about malaria transmission and its preventive measures among households in urban areas of Assosa Zone, Western Ethiopia. Ethiopian Journal of Health Development. 2007, 21(2): 157–165. Available online at ISSN 1021-6790.

[10] Kendie FA, Nibret Semegn E, Ferede MW. Prevalence of malaria among adults in Ethiopia: a systematic review and meta-analysis. Journal of tropical medicine. 2021;2021. Available online at https://dx.doi.org/10.1155%2F2021%2F8863002. doi: 10.1155/2021/8863002 33747096 PMC7952180

[11] Ethiopia Ministry of Health, National Malaria Guidelines, 4th edition. Addis Ababa, Ethiopia: Ethiopian Federal Ministry of Health. 2017. Available online at (Google Scholar).

[12] Federal Democratic Republic of Ethiopia Ministry of Health. *National Malaria Guidelines*. 2017. Available online (Google Scholar).

[13] Cadena B, N D, and Vittor A. Deforestation and vector-borne disease: forest conversion favors important mosquito vectors of human pathogens. Basic and applied ecology. 2018; 26: 101–110. Available online at doi: 10.1016/j.baae.2017.09.012 34290566 PMC8290921

[14] Alemu A, Tsegaye W, Golassa L, and Abebe G. Urban malaria and associated risk factors in Jimma town, south-west Ethiopia. Malaria journal. 2011; 10(1): 1–10. Available online at http://www.malariajournal.com/content/10/1/173. doi: 10.1186/1475-2875-10-173 21699741 PMC3128012

[15] Girum T, Shumbej T, and Shewangizaw M. *Shewangizaw*, Burden of malaria in Ethiopia, 2000–2016: findings from the Global Health Estimates 2016. Tropical Diseases, Travel Medicine and Vaccines. 2019; 5(1): 1–7. Available online at doi: 10.1186/s40794-019-0090-z 31338202 PMC6626392

[16] World Health Organization, World malaria report 2015. Available online at ISBN 978 92 4 069443 9(pdf).

[17] Berhe B, Mardu F, Legese H, and Negash H. Seasonal distribution and seven-year trend of malaria in North West Tigrai: 2012–2018, Ethiopia; *2019*. Tropical diseases travel medicine and vaccines. 2019; 5(1): 1–7. Available online at doi: 10.1186/s40794-019-0091-y 31428440 PMC6694521

[18] Deressa W, Ali A, and Berhane Y. Review of the interplay between population dynamics and malaria transmission in Ethiopia. Ethiopian Journal of Health Development. 2006; 20(3). Available online doi: 10.4314/ejhd.v20i3.46823

[19] Ethiopian Public Health Institute, National Research Institute, and Federal Minister of Health. Ethiopia national malaria indicator survey 2015. Federal Ministry of Health Addis Ababa. https://www.malariasurveys.org/documents/Ethiopia_MIS_2015.pdf.

[20] Belete E, and Roro A. Malaria prevalence and its associated risk factors among patients attending Chichu and Wonago Health Centres, South Ethiopia. Journal of Research in Health Sciences. 2016. 16(4): p. 185.19. www.umsha.ac.ir/jrhs. 28087849 PMC7189928

[21] Bidu K, and Babure Z. Prevalence of Malaria and Associated Factors among Febrile Patients Visiting Kalala Health Center in Haro Limmu Woreda, East Wollega Zone, Western Ethiopia, 2016. Epidemiology (Sunnyvale). 2019; 9(365): 2161–1165. Available online at doi: 10.4172/2161-1165.1000365

[22] Woyessa A., Deressa W, Ali A, and Lindtjørn B. Prevalence of malaria infection in Butajira area, south-central Ethiopia. Malaria journal. 2012; 11(1): 1–8. Available at http://www.malariajournal.com/content/11/1/84. doi: 10.1186/1475-2875-11-84 22443307 PMC3383546

[23] Boadu I, Nsemani W, Ubachukwu P, and Okafor F. Knowledge and Prevalence of Malaria among Rural Households in Ghana. Journal of Community Medicine Health Education. 2020; 10(673): 2. Available online at ISSN: 2161-0711.

[24] Sultana M, Sheikh N, Mahumud R, Jahir T, Islam Z, and Sarker A. Prevalence and associated determinants of malaria parasites among Kenyan children. Tropical Medicine and Health. 2017;45(1):1–9. Available online at doi: 10.1186/s41182-017-0066-5 29085254 PMC5651573

[25] Umaru M, and Uyaiabasi G. Prevalence of malaria in patients attending the general hospital Makarfi, Makarfi Kaduna–State, North-Western Nigeria. American journal of infectious Diseases and Microbiology. 2015; 3(1): 1–5. Available online at doi: 10.12691/ajidm-3-1-1

[26] Dayanand K, Punnath K, and Chandrashekar V. et al., Malaria prevalence in Mangaluru city area in the southwestern coastal region of India. Malaria journal. 2017; 16(1): 1–10. 29258505 10.1186/s12936-017-2141-0 PMC5735873

[27] Ramdzan A, Ismail A., and Zanib Z.M., Prevalence of malaria and its risk factors in Sabah, Malaysia. International Journal of Infectious Diseases. 2020; 91: 68–72. Available online at www.elsevier.com/locate/ijid. doi: 10.1016/j.ijid.2019.11.026 31785400

[28] Jenkins R, Omollo R, Ongecha M. Prevalence of malaria parasites in adults and its determinants in malaria endemic area of Kisumu County, Kenya. Malaria journal. 2015; 14(1): 1–6. Available online at doi: 10.1186/s12936-015-0781-5 26152272 PMC4495611

[29] Rulisa S, Kateera F, Bizimana J. Malaria prevalence, spatial clustering and risk factors in a low endemic area of Eastern Rwanda: a cross-sectional study. PloS one. 2013; 8(7). Available online at doi: 10.1371/journal.pone.0069443 23936018 PMC3720654

[30] Tadesse F, Fogarty A, and Deressa W. Prevalence and associated risk factors of malaria among adults in East Shewa Zone of Oromia Regional State, Ethiopia: a cross-sectional study. BMC Public Health. 2017; 18(1): 25. Available online at doi: 10.1186/s12889-017-4577-0 28716009 PMC5513396

[31] Abossie A, Yohanes T, Nedu A, Tafesse W, and Damitie M. Prevalence of malaria and associated risk factors among febrile children under five years: A cross-sectional study in Arba Minch Zuria district, south Ethiopia. Infection and drug resistance. 2020; 13: 363. Available online at doi: 10.2147/IDR.S223873 32104008 PMC7012238

[32] Tarekegn M, Tekie H, Dugassa S, and Wolde H Y. Malaria prevalence and associated risk factors in Dembiya district, North-western Ethiopia. Malaria Journal. 2021; 20(1): 1–11. 34535130 10.1186/s12936-021-03906-9 PMC8447688

[33] Belay B, Tegenu G, and Araya G. Malaria Prevalence and Knowledge, Attitude and Practice about Malaria among Febrile Patients Attending Chagni Health Center. Northwest Ethiopia: A Cross-Sectional Study.2020. doi: 10.21203/rs.3.rs-27951/v1 PMC8256592 34218813

