# Supplementary material for: Malaria prevalence and risk factors among patients visiting Mizan Tepi University Teaching Hospital, Southwest Ethiopia
Source: PLoS One. 2022 Jul 28;17(7):e0271771. doi: 10.1371/journal.pone.0271771 (PMC9333314; doi:10.1371/journal.pone.0271771)
Supplement: S1 File — (DOCX) [file pone.0271771.s002.docx]

**Malaria prevalence and risk factors among patients visiting Mizan Tepi University Teaching Hospital in Southwest Ethiopia**

Tadesse Duguma **^1^***, Eyob Tekalign **^1&^**, Dassalegn Muleta **^2&^**, Asnake Simieneh **^2&^**

**^1,2^**Department of Medical Laboratory Science, College of Health Science and Medicine, Mizan-Tepi University, Mizan-Aman, Ethiopia

*Corresponding author

E-mail: [tadesse.dhuguma@gmail.com(TD)](mailto:tadesse.dhuguma@gmail.com(TD))

&These authors contributed equally to this work.

**Abstract**

**Background:** Ethiopia is among sub-Saharan African countries with a high number of malaria cases each year, with most of the landmass favoring the breeding of the vectors. There have been extensive efforts to control and prevent the transmission of malaria, which is part of the country's prevention-based health policy.

**Objective:** This study aimed to determine malaria prevalence and associated risk factors among patients visiting Mizan-Tepi University Teaching Hospital (MTUTH).

**Materials and methods:** A cross-sectional study was conducted from September to December 2021 among patients visiting MTUTH, Southwest Ethiopia. A pretested structured questionnaire was used to collect sociodemographic data, and a capillary blood sample was collected after obtaining written informed consent from the study participants. The data were entered into Epi-data manager (v4.0.2.101) and analyzed with SPSS version 25.0, with a P-value of < 0.05 set as a significance.

**Results:** A total of 439 patients participated, of which 91 (20.7%) were positive for malaria parasites, with a higher prevalence observed among the age group interval of 25–34 years (5.5%). Patients living in areas of stagnant water were more likely to get infected with the malaria parasite (AOR = 16.191, 95% CI: 9.137, 28.692) compared to those who live away, and individuals living in houses not sprayed with insecticides were more susceptible to malaria infection (AOR = 0.215, 95% CI: 0.128, 0.360).

**Conclusion:** The overall malaria prevalence in this study was 91 (20.7%), which proves that malaria remains a major threat to the communities in the study area, with *Plasmodium falciparum* contributing to most of the cases. Improving the habits of ITN usage and indoor residual spray through health education may help to reduce the impact of malaria in the study area.

**Keywords:** malaria prevalence, associated risk factors, Mizan, Southwest Ethiopia **Introduction**

Malaria is a major public health problem that still results in illness and death. Globally, malaria cases showed an increasing pattern from 227 million cases in 2019 to 241 million in 2020, which was reported from 85 malaria-endemic countries [1], with most of this increase coming from countries in the WHO African Region [1]. The WHO African regions contributed to about 95% of malaria cases worldwide, and in recent years, malaria has shown a rise of 14 million more cases and 47,000 more deaths than what was reported across the globe previously, due to disruptions to services during the coronavirus pandemic [2]. Malaria is one of the major diseases affecting poor people in developing countries. The majority of the global malaria burden is in sub-Saharan Africa, with the highest global cases and deaths that can be observed from these regions, which accounted for the majority (95%) of these cases. Nigeria (27%), the Democratic Republic of the Congo (12%), Uganda (5%), Mozambique (4%), and Niger (3%) accounted for about 51% of all cases globally [3]. It hurts people's health as well as the economic development of many developing countries, particularly in sub-Saharan Africa [1, 4, 5]. Ethiopia, as one of those countries, suffers a lot from this disease, which is posing a problem to the health and economy of the country. The distribution of malaria in Ethiopia varies depending on climate, rainfall patterns, and altitude [6]. Regional subgroup analysis showed that the highest malaria prevalence was recorded in the Southern Nations, Nationalities, and Peoples' Region (SNNPR) (16.17%), followed by Oromia Regional State (13.11%), and Amhara Regional State (12.41%) [7]. The most common causes of human malaria are four species, namely, *Plasmodium vivax, Plasmodium falciparum, Plasmodium malariae*, *Plasmodium ovale*, and sometimes a fifth species, *Plasmodium knowlesi*, which is a monkey parasite [8]. The most prevalent and pathogenic malaria parasite, most commonly associated with severe illness and death, accounting for 99.7% of malaria cases in the WHO African region, 70.9% from Central America, 20.8% from South America, and 4.8% from Asia, is *P. falciparum* [9]. *Plasmodium falciparum* is responsible for most human infections, but *Plasmodium vivax* is the most widely distributed species due to the geographic reach of the parasite [10]. *P. vivax* is the only one of the four species that extends through tropical, subtropical, and temperate regions. *P. falciparum* is continuing to cause infection in the tropical and sub-tropical regions and is probably the worst cause of a lethal form of malaria [11].

Ethiopia is one of the few African countries where both *Plasmodium falciparum* and *Plasmodium vivax* species are co-endemic in substantial proportions [12]. The *Anopheles* mosquito serves as the primary vector for malaria transmission in Ethiopia. *Anopheles arabiensis* is the main vector, followed by *Anopheles phronesis, Anopheles funestus*, and *Anopheles nili*, which play a role as secondary vectors [13]. The risk of infection is determined by the number and species of mosquitoes present in a given area and the climate and geography [14].

Ethiopia has three climatic zones, namely "Kola", "Woina Dega", and "Dega", of which the first two have favorable characteristics (i.e., warmer and humid climate conditions) for malaria endemicity [15]. The "Kola," or hot zone below 1500 meters altitude, has seasonal malaria transmission with moderate to high endemicity (46% of the territory), whereas the "Woina Dega," or temperate zone, has malaria transmission characterized by sporadic outbreaks of unstable malaria caused by sudden climatic changes such as heavy rain or clouds. The "Dega," or cold zone (8% of the territory), is a climatic area above 2500 meters altitude where malaria transmission is not common [15]. Nearly 75% of the country's landmass is conducive to malaria transmission, putting a large portion of the population at a 68% risk of contracting the disease, and malaria is estimated to kill 70,000 people each year [16,17].

In Ethiopia, malaria transmission varies from one season to another due to variation in the altitude of areas and a relatively longer duration of transmission of the disease in lowland areas, river basins, and valleys [18]. There are two malaria transmission seasons during which the vectors are abundant; that is from September to December and the early rainy season of April to May, which overlaps with the major harvesting seasons [18]. Malaria outbreaks are common in some of Ethiopia's highland or highland fringe areas, mainly 1,000 to 2,000 meters above sea level [19]. Climate changes include alternations in one or more climate variables, including temperature, precipitation, wind, and sunshine [17, 20]. 2.9 million malaria cases and 4,782,000 related mortalities have been reported per year, and morbidity and mortality rates dramatically increase during epidemics [16]. Despite key achievements and progress in reducing the burden of the disease, malaria remains a major health problem for the public. It is among the 10 top leading causes of illness and death in large segments of the population, including children under the age of five and adults [21]. Malaria is also ranked at the top of hospital admissions, outpatient visits, and mortalities [21]. It is important to screen and diagnose patients on time and treat them immediately to prevent further transmissions [20]. Ethiopia is still one of the countries with a high malaria burden, and this was evidenced by the deaths of people in the study area, including children and pregnant women, which resulted in reduced working capacity and other day-to-day activities of the community. As a result, this study may provide new insight or could serve as baseline data for the concerned bodies to boost preventative and control methods to reduce and, in the long run, eliminate the disease's health impact.

**Materials and methods**

**Ethical statement**

Ethical approval was obtained from the research committee of the college of health sciences and medicine of Mizan-Tepi University before the commencement of the study. Written informed consent was obtained from all participants except for infants, children, and minors, whose consent was obtained through their parents/guardians after explaining the study's purpose, possible risks, and benefits. Moreover, participants were assured that participation is entirely voluntary and can be withdrawn at any time during the data collection process.

**Study area and period**

The study was conducted between September and December 2021 at Mizan-Tepi University Teaching Hospital in Mizan-Aman town, Benchi-Sheko zone, SNNPR region, Southwest Ethiopia, which is located 565 kilometers from Addis Ababa, the capital of the country. The research site is located in a region of the country known for its warm and humid climate, with coordinates of 7°0′N 35°35′E and an elevation of **1451** meters. Based on the 2012 census conducted by the central statistical agency, the total population of Mizan-Aman town was 63,193, of whom 32,596 were male and 30,596 were female. Mizan-Aman town is the administrative town of the Benchi-Sheko zone, one of 13 zones of the Southern Nations Nationalities Peoples Regional State, and has 12 woredas (districts).

**Study design and population:**

An institutional-based cross-sectional study was employed, including all patients who visited MTUTH during the study period as the source population, while all malaria-suspected patients who were sent to the laboratory of the hospital for blood film examination were taken as a study population, whereas those willing to participate and who were volunteers to provide blood samples were recruited as the study subjects. Anyone who took antimalarial drugs and antibiotics in the last month was not included in the study.

**Sample size and sampling technique**

The sample size was calculated using a single population proportion formula, and the following assumptions were considered at a 95% confidence interval, an estimated malaria prevalence rate of 11% (based on a study from Jimma zone, Southwest Ethiopia) [15]. Based on these considerations, the sample size was calculated using the following formula,

= (1.96)2 0.11(1-0.11)/0.032= 417.880≈418

After adding a 10% buffer for possible non-response,

the final sample size became 418 + 418(0.1) =459.8≈460

Where n= minimum sample size

P= estimated malaria prevalence rate of 11% (study from the Jimma Zone, Southwest Ethiopia)

d = error margin (3%)

(Zα/2) )2 = the standard normal variable.

The study participants were recruited by a systematic random sampling technique, considering the case flow in the hospital from September to December of the previous year. **Data collection instruments and procedures**

Structured questionnaires adapted from related literature [22], that contain both socio-demographic and risk factor variables were used to collect the data. Before blood sample collection, the finger was cleaned with 70% alcohol-moistened cotton. A drop of blood, approximately 50 μL (capillary blood from fingertip) by finger prick, was collected from each study subject, and both thick and thin films were made according to the standard operating procedure.

**Data collection process and management**

Before the start of the data collection process, two-day training on the objective of the study was given to the data collectors (interview and blood sample), and written informed consent was obtained from the study participants (adults); and for infants, children, and minors, consent was obtained by communicating with their guardian/caregiver, or parents. Three medical laboratory technicians were recruited for data collection, along with two supervisors to facilitate the data collection processes. The blood films (thick and thin) were stained using a 10% Giemsa working solution and examined microscopically using a 100X oil immersion objective to detect malaria parasites.

**Data quality assurance**

The data collection procedures, tools used, and how to handle ethical issues were discussed with the data collectors. A pretest was conducted on 5% of the sample size before the commencement of the main study in Tepi general hospital, which is located 50 kilometers away from the study area. The questionnaire was translated into the respondent’s language during data collection. Regular supervision by the supervisors and the principal investigators was conducted to ensure that all the necessary data was properly collected.

**Statistical analysis**

The Epi-data manager (v4.0.2.101) was used to enter data, and SPSS version 25.0 was used for analysis. Descriptive statistics and both bivariate and multivariable logistic regression were performed to assess the existence of an association between the outcome variable and the risk factors.

**Results and discussion**

**Socio-demographic characteristics of the study subjects**

Of the total 439 patients who participated in this study, a significant number of participants (91) were infected with malaria parasites. This study involves 208 males and 231 females, and the malaria prevalence was found to be almost similar for both. Malaria was reported in all age groups, but the infection rate was highest in the 25–34 age group (5.5%). Almost half of the study participants were illiterates (i.e., people who had no formal education). Of the different occupations of those included in the study, the most cases were among housewives (35) and students (22) [Table 1].

**Table 1****: Malaria prevalence by sex, age, educational level, and occupation among patients undergoing blood film examination at MTUTH in 2021 (n**$\boldsymbol{=}$**439)**

| **Socio-demographic variables** | | **Malaria** | | |
| --- | --- | --- | --- | --- |
|  |  | **Positive**  **N (%)** | **Negative**  **N (%)** | **Total**  **N (%)** |
| Sex | Male | 43(9.8) | 165(37.6) | 208(47.4) |
|  | Female | 48(10.9) | 183(41.7) | 231(52.6) |
| Age  in year/s | 0-4 | 6(1.4) | 49(11.2) | 55(12.5) |
|  | 5-14 | 11(2.5) | 63(14.4) | 74(16.9) |
|  | 15-24 | 15(3.4) | 76(17.3) | 91(20.7) |
|  | 25-34 | 24(5.5) | 86(19.6) | 110(25.1) |
|  | 35-44 | 20(4.6) | 49(11.2) | 69(15.7) |
|  | 45-55 | 13(3) | 23(5.2) | 36(8.2) |
|  | >55 | 2(0.5) | 2(0.5) | 4(0.9) |
| Educational  status | Illiterate | 53(12.1) | 166(37.8) | 219(49.9) |
|  | Literate | 38(8.6) | 182(41.5) | 220(50.1) |
| Occupational  status | Farmer | 10(2.3) | 41(9.3) | 51(11.6) |
|  | Merchant | 0(0) | 15(3.4) | 15(3.4) |
|  | Government Employee | 7(1.6) | 29(6.6) | 36(8.2) |
|  | Student | 22(5) | 113(25.7) | 135(30.8) |
|  | Housewife | 35(8) | 89(20.3) | 124(28.2) |
|  | Daily worker | 15(3.4) | 45(10.3) | 60(13.7) |
|  | Other | 2(0.5) | 16(3.6) | 18(4.1) |
| **Total** |  | 91(20.7) | 348(79.3) | 439(100) |

**Malaria prevalence**

A total of 460 study participants were recruited for this study, of which only 439 participated in the interview and provided blood samples (a non-response rate of 4.6%). Ninety-one (20.7%) of those tested were found to be infected with malaria parasites, of which 53 (12.1%), 33 (7.5%), and 5 (1.1%) were *Plasmodium falciparum, Plasmodium vivax*, and mixed infection, respectively [Fig 1].

**Fig 1: Plasmodium species and their distribution.**

**Malaria prevalence in relation to climate conditions**

Of the total study participants, 349 (79.5%), 68 (15.5%), and 22 (5.0%) were from warmer "Kolla" conditions, while medium "Woina Dega" and colder "Dega" conditions had a respective malaria prevalence of (17.8%), (2.3%), and (0.7%), respectively.

**Possible risk factors for malaria infection**

Data was collected from 439 study participants to investigate the contributions of possible risk factors to the prevalence of malaria. The presence of stagnant water near the residence, a house sprayed with insecticide, a history of anti-malarial treatment, residence type, and last night's ITN usage were among the possible risk factors assessed. Accordingly, more than three-fourths of respondents did not have access to insecticide-treated bed nets (ITNs), and more than two-thirds of the participants reported that their houses were not sprayed with indoor residual spray, indicating a lack of access to ITNs and inadequate insecticide spray contributed to the malaria prevalence observed in this study. This study showed not only inadequate access to ITNs but also a low level of ITN usage at 90 (20.5%). Some risk factors were associated with malaria infection (AOR = 16.191, 95% CI: 9.137, 28.692), (AOR = 1.799, 95% CI: 1.054, 3.073), (AOR = 0.215, 95% CI: 0.128, 0.360), and (AOR = 1.971, 95% CI: 1.034, 3.756), for presence of stagnant water near residential, house sprayed with insecticide, history of anti-malarial treatment and residence respectively, but not with access to and last night's ITN usage [Table 2].

**Table 2: Bivariate and multivariable analysis of factors associated with malaria infection concerning ITN availability, ITN usage, presence of stagnant water, indoor residual spray, history of malaria treatment, and residence at MTUTH, 2021 (n = 439)**

| **Variables** | | **Malaria** | | | **OR (95% CI)** | |
| --- | --- | --- | --- | --- | --- | --- |
|  |  | **Positive**  **N (%)** | **Negative**  **N (%)** | **Total**  **N (%)** |  |  |
|  |  |  |  |  | **COR** | **AOR** |
| ITN Availability | Yes | 15(3.4) | 90(20.5) | 105(23.9) | 1.00 | 1.00 |
|  | No | 76(17.3) | 258(58.8) | 334(76.1) | 0.566(0.309,1.035) | 1.949(0.972,3.907) |
| Last night's ITN usage | Yes | 17(3.8) | 73(16.6) | 90(20.5) | 1.00 | 1.00 |
|  | No | 74(16.9) | 275(62.7) | 349(79.5) | 0.865(0.481,1.556) | 0.821(0.415,1.622) |
| Presence of Stagnant water | Yes | 72(16.4) | 66(15) | 138(31.4) | 0.062(0.035,0.109) * | 16.191(9.13,28.692) ***** |
|  | No | 19(4.3) | 282(64.2) | 301(68.6) | 1.00 | 1.00 |
| House sprayed with insecticide | Yes | 21(4.8) | 122(27.8) | 143(32.6) | 1.00 | 1.00 |
|  | No | 70(15.9) | 226(51.5) | 296(67.4) | 0.556(0.325,0.949) * | 1.799(1.054,3.073) * |
| History of anti-malarial treatment | Yes | 43(9.8) | 55(12.5) | 98(22.3) | 1.00 | 1.00 |
|  | No | 48(10.9) | 293(66.7) | 341(77.7) | 4.772(2.888,7.887) * | 0.215(0.128,0.360) * |
| Residence | Urban | 30(6.8) | 193(44.0) | 223(50.8) | 1.00 | 1.00 |
|  | Rural | 61(13.9) | 155(35.3) | 216(49.2) | 0.395(0.243, 0.642) * | 1.971(1.034,3.756) * |
| Total |  | 91(20.7) | 348(79.3) | 439(100) |  | |

**Abbreviations**: AOR, adjusted odds ratio; COR, crude odds ratio; OR, odds ratio, (*) indicates significance at p<0.05, "1.00 represents" reference category during analysis

Of the total participants in this study, ninety-one (20.7%) of them were infected with malaria parasites, with the highest prevalence due to *Plasmodium falciparum* (12.1%), followed by *Plasmodium vivax* (7.5%). A study from Butajira, south-central Ethiopia, found a similar prevalence of *Plasmodium falciparum* (12.4%) [23]. The findings from this study revealed that the prevalence of malaria was higher among females (10.9%) than males (9.8%), which is in line with a previous study conducted in Ghana [24]. In this study, the prevalence of malaria was found to be higher in the 25–34 age group (5.5%) compared to other age groups, which is in line with a previous study conducted in Ghana that reported the prevalence of malaria to be higher in similar age groups [24]. The prevalence of malaria among illiterates was higher (12.1%) compared to those with formal education (8.6%). The reason for these variations may be due to differences in the level of understanding of the preventive and control methods among the study participants. Patients with different occupations participated in this study, in which 35 (8%) housewives and 22 (5%) students were diagnosed with malaria. Out of the positive cases, 226 (51.5%) study participants responded that their house was not sprayed with insecticide/chemical compared to those whose house was sprayed (4.8%), which may explain the relatively high malaria prevalence (15.9%) in this study. Malaria prevalence was found to be higher in rural residents (13.9%) as compared to urban residents (6.8%). This could be due to the expected low exposure and accessibility of the media for communication in rural communities. Although the study participants agreed that "the usage of it is a powerful vector control tool for the prevention of malaria transmission and hence reduces the prevalence of the disease elsewhere in the country where malaria is endemic", only 23.9% of them had ITNs in their houses, and ITN ownership by itself is not a guarantee of its usage, which is revealed by the result that showed more than three-fourths (79.5%) of the study participants did not use their bed nets. See [Table 2]. Insecticide bed net use in this study was similar to what was found in a study in Kenya: approximately 92.11% of mosquito bed net usage, and malaria prevalence was observed to be lower among households that used ITNs (8.05%) than those that did not use them (23.11%). [25]. The use of ITNs is one of the most effective malaria prevention methods. The prevalence of malaria among those who were not using ITNs (74, 16.9%) was higher as compared to those who used them (17, 3.8%). More than half of the study participants responded that they had no ITN in their houses, which is evidenced by the high malaria infection observed among those ITN non-users. Seventy-two (16.4%) patients whose blood film examination revealed the presence of malaria parasites responded that there was stagnant water near their homes. The malaria prevalence found in this study (20.7%, 91/439) was lower as compared to studies conducted in different parts of the world, including various areas of Nigeria (35.7%, 419/1173) [26], India (36.6%) [27], Malaysia (33.6%, 410/1222) [28], and Kenya (28%, 325/1158) [29]. It is also comparable to findings from Rwanda (22.8%, 175/769) [30], East Shewa Ethiopia (20.5%, 170/830) [31], Arba Minch South Ethiopia (22.1%, 60/271) [32], and much higher compared to studies from North-West Ethiopia (7.3%, 296/4077), (3.5%, 26/735) [33, 34]. These variations may be due to the differences in the geographical location and climate conditions of the study areas. The result of this study showed that malaria is still a serious public health concern in different locations of the country, so the information obtained from this particular study can be used to devise means (control and prevention strategies) to prevent further suffering of people from this disease.

**Limitations**

The findings of this study could have been better if testing had been done using advanced molecular techniques like polymerase chain reaction (PCR) and loop-mediated isothermal amplification (LAMP), which have higher detection abilities compared to light microscopy.

**Conclusions**

The overall malaria prevalence in the study was 91 (20.7%), which proves that malaria remains a major health problem in the area with *Plasmodium falciparum* as the predominant species in the study area. Improving the habits of ITN usage in the community through health information dissemination may help to prevent the transmission.

**Lists of abbreviations**

AOR adjusted odds ratio

COR crude odds ratio

ITNs insecticide-treated nets

LAMP loop-mediated isothermal amplification

OR Odds ratio

PCR Polymerase chain reaction

SPSS Statistical packages for social sciences

WHO World health organization

**Acknowledgments**

The authors are very thankful for the Mizan-Tepi University Teaching Hospital administrative bodies and staff for their contributions, the study participants for their willingness to take part in the study, the data collectors, and the supervisors for their close follow-up during the data collection process.

**References**

1. World Health Organization. "World Malaria Report 2020". 20 years of global progress and challenges, 2020. Available <https://www.who.int/publications-detail-redirect/97892400157919789240015791-eng.pdf>

2. World Health Organization, "World Malaria Report 2021". Available online at ISBN 978-92-4-004049-6 (electronic version).

3. Badmos AO, Alaran AJ, Adebisi YA, Bouaddi O, Onibon Z, Dada A, and others.sub-Saharan African countries can learn from malaria elimination in China. Tropical Medicine and Health. 2021;49(1):1-6. Available online at https://doi.org/10.1186/s41182-021-00379-z

4. Barofsky J, Claire C, Tobenna A, and Farshad F. The economic effects of malaria eradication: Evidence from intervention in Uganda: Program on the Global Demography of Aging Working Paper. 2011; (70). Available online at <http://www.hsph.harvard.edu/pgda/working.htm>

5. Castillo R, McIntyre M, and Barnes KD. The household burden of malaria in South Africa and Mozambique: is there a catastrophic impact? Tropical medicine & international health. 2008; **13**(1): 108-122. Available online at [https://doi:10.1111/j.1365-3156.2007.01979.x](doi:10.1111/j.1365-3156.2007.01979.x)

6. Legesse Y, Ayalew T, Tefera B, and Kora T. Knowledge, attitude, and practice about malaria transmission and its preventive measures among households in urban areas of Assosa Zone, Western Ethiopia*.* Ethiopian Journal of Health Development, 2007, **21**(2): 157-165. Available online at [ISSN 1021-6790](file:///C:\Users\Administrator\AppData\Roaming\Microsoft\Word\1021-6790)

7. Kendie FA, Nibret Semegn E, Ferede MW. Prevalence of malaria among adults in Ethiopia: a systematic review and meta-analysis. Journal of tropical medicine. 2021;2021. Available online at <https://dx.doi.org/10.1155%2F2021%2F8863002>

8. Krief S, Pacheco AE, Mugisha M A. et al. On the diversity of malaria parasites in African apes and the origin of *Plasmodium falciparum* from Bonobos*.* PLoS pathogens,2010; **6**(2). Available online at [https://doi:10.1371/journal.ppat.e1000765](doi:10.1371/journal.ppat.e1000765)

9. Mace KE, Arguin PM, Tan KR. Malaria surveillance United States, 2015. MMWR Surveillance Summaries. 2018;67(7):1. Available online at https://doi.org/10.15585/mmwr.ss6707a1

10. World Health Organization. World malaria report: Geneva. 2018. Available online at (Google Scholar)

11. Dress T, and Girma M. *Plasmodium falciparum* and *Plasmodium vivax* prevalence in Ethiopia: a systematic review and meta-analysis*.* Malaria research and treatment, 2019. **2019**. Available online at <https://doi.org/10.1155/2019/7065064>

12. Ethiopia Ministry of Health, National Malaria Guidelines, 4th edition. Addis Ababa, Ethiopia: Ethiopian Federal Ministry of Health, 2017. Available online at (Google Scholar).

13. Federal Democratic Republic of Ethiopia Ministry of Health. *National Malaria Guidelines.* 2017. Available online (Google Scholar).

14. Cadena B, D N, and Vittor A. Deforestation and vector-borne disease: forest conversion favors important mosquito vectors of human pathogens*.* Basic and applied ecology, 2018; **26**: 101-110. Available online at [https://doi: 10.1016/j.baae.2017.09.012](doi:%2010.1016/j.baae.2017.09.012)

15. Alemu A, Tsegaye W, Golassa L, and Abebe G. Urban malaria and associated risk factors in Jimma town, south-west Ethiopia. Malaria journal. 2011; **10**(1): 1-10. Available online at <http://www.malariajournal.com/content/10/1/173>

16. Girum T, Shumbej T, and Shewangizaw M. *Shewangizaw,* Burden of malaria in Ethiopia, 2000-2016: findings from the Global Health Estimates 2016*.* Tropical Diseases, Travel Medicine and Vaccines, 2019; **5**(1): 1-7. Available online at <https://doi.org/10.1186/s40794-019-0090-z>

17. World Health Organization, World malaria report 2015. Available online at [ISBN 978 92 4 069443 9](file:///C:\Users\Administrator\AppData\Roaming\Microsoft\Word\ISBN%20%20978%2092%204%20069443%209)(pdf)

18. Berhe B, Mardu F, Legese H, and Negash H. Seasonal distribution and seven-year trend of malaria in North West Tigrai: 2012–2018, Ethiopia*; 2019.* Tropical diseases travel medicine and vaccines. 2019; **5**(1): 1-7. Available online at <https://doi.org/10.1186/s40794-019-0091-y>

19. Deressa W, Ali A, and Berhane Y. Review of the interplay between population dynamics and malaria transmission in Ethiopia*.* Ethiopian Journal of Health Development, 2006; **20**(3). Available online [DOI: 10.4314/ejhd.v20i3.46823](DOI:%2010.4314/ejhd.v20i3.46823)

20. Ethiopian Public Health Institute, National Research Institute, and Federal Minister of Health. Ethiopia national malaria indicator survey 2015. Federal Ministry of Health Addis Ababa. Available online https://www.malariasurveys.org/documents/Ethiopia_MIS_2015.pdf

21. Belete E, and Roro A. Malaria prevalence and its associated risk factors among patients attending Chichu and Wonago Health Centres, South Ethiopia. Journal of Research in Health Sciences, 2016. 16(4): p. 185.19. Available online at [www.umsha.ac.ir/jrhs](file:///C:\Users\Administrator\AppData\Roaming\Microsoft\Word\www.umsha.ac.ir\jrhs)

22. Bidu K, and Babure Z. Prevalence of Malaria and Associated Factors among Febrile Patients Visiting Kalala Health Center in Haro Limmu Woreda, East Wollega Zone, Western Ethiopia, 2016*.* Epidemiology (Sunnyvale), 2019; **9**(365): 2161-1165. Available online at [https://doi: 10.4172/2161-1165.1000365](doi:%2010.4172/2161-1165.1000365)

23. Woyessa A., Deressa W, Ali A, and Lindtjørn B. Prevalence of malaria infection in Butajira area, south-central Ethiopia. Malaria journal. 2012; **11**(1): 1-8. Available at [http://www.malariajournal.com/content/11/1/84](http://www.malariajournal.com/content/11/1/84%20)

24. Boadu I, Nsemani W, Ubachukwu P, and Okafor F. Knowledge and Prevalence of Malaria among Rural Households in Ghana*.* J Community Med Health Educ, 2020; **10**(673): 2. Available online at [ISSN: 2161-0711](ISSN:%202161-0711)

25. Sultana M, Sheikh N, Mahumud R, Jahir T, Islam Z, and Sarker A. Prevalence and associated determinants of malaria parasites among Kenyan children*.* Tropical Medicine and Health. 2017;**45**(1):1-9. Available online at https://doi 10.1186/s41182-017-0066-5

26. Umaru M, and Uyaiabasi G. Prevalence of malaria in patients attending the general hospital Makarfi, Makarfi Kaduna–State, North-Western Nigeria. American journal of infectious Diseases and Microbiology. 2015; **3**(1): 1-5. Available online at [https;//doi:10.12691/ajidm-3-1-1](doi:10.12691/ajidm-3-1-1)

27. Dayanand K, Punnath K, and Chandrashekar V. et al., Malaria prevalence in Mangaluru city area in the southwestern coastal region of India. Malaria journal. 2017; **16**(1): 1-10. Available online at <https://doi.org/10.1186/s12936-017-2141-0>

28. Ramdzan A, A. Ismail, and Z.M. Zanib, Prevalence of malaria and its risk factors in Sabah, Malaysia. International Journal of Infectious Diseases. 2020; **91**: 68-72. Available online at [*www.elsevier.com/locate/ijid*](file:///C:\Users\Administrator\AppData\Roaming\Microsoft\Word\www.elsevier.com\locate\ijid)

29. Jenkins R, Omollo R, Ongecha M. Prevalence of malaria parasites in adults and its determinants in malaria endemic area of Kisumu County, Kenya*.* Malaria journal, 2015; **14**(1): 1-6. Available online at [https://doi 10.1186/s12936-015-0781-5](file:///C:\Users\Administrator\AppData\Roaming\Microsoft\Word\DOI%2010.1186\s12936-015-0781-5)

30. Rulisa S, Kateera F, Bizimana J. Malaria prevalence, spatial clustering and risk factors in a low endemic area of Eastern Rwanda: a cross-sectional study. PloS one. 2013; **8**(7). Available online at [https://doi:10.1371/journal.pone.0069443](doi:10.1371/journal.pone.0069443)

31. Tadesse F, Fogarty A, and Deressa W. Prevalence and associated risk factors of malaria among adults in East Shewa Zone of Oromia Regional State, Ethiopia: a cross-sectional study. BMC public health. 2018; 18(1):1-8.Tadesse et al. BMC Public Health (2018) 18:25. Available online at [http://doi 10.1186/s12889-017-4577-0](file:///C:\Users\Administrator\AppData\Roaming\Microsoft\Word\DOI%2010.1186\s12889-017-4577-0)

32. Abossie A, Yohanes T, Nedu A, Tafesse W, and Damitie M. Prevalence of malaria and associated risk factors among febrile children under five years: A cross-sectional study in Arba Minch Zuria district, south Ethiopia. Infection and drug resistance. 2020; 13: 363. Available online at <http://doi.org/10.2147/IDR.S22387>

33. Tarekegn M, Tekie H, Dugassa S, and Wolde‑H Y. Malaria prevalence and associated risk factors in Dembiya district, North-western Ethiopia. Malaria Journal. 2021; **20**(1): 1-11. Available online at <https://doi.org/10.1186/s12936-021-03906-9>

34. Belay B, Tegenu G, and Araya G. Malaria Prevalence and Knowledge, Attitude and Practice about Malaria among Febrile Patients Attending Chagni Health Center. Northwest Ethiopia: A Cross-Sectional Study.2020. Available online at <https://doi:10.21203/rs.3.rs-27951/v1>

**Supporting information**

S1 Data set. (SAV)

S1 File. (DOCX)
